# Lymphocyte apoptosis in murine *Pneumocystis *pneumonia

**DOI:** 10.1186/1465-9921-10-57

**Published:** 2009-06-26

**Authors:** Xin Shi, Nicole J LeCapitaine, Xiaowen L Rudner, Sanbao Ruan, Judd E Shellito

**Affiliations:** 1 Section of Pulmonary/Critical Care Medicine, LSU Health Sciences Center, New Orleans, LA 70112, USA

## Abstract

**Background:**

Apoptosis of lymphocytes is important in the termination of an immune response to infection but has also been shown to have detrimental effects in animal models of systemic infection and sepsis. We sought to characterize lymphocyte apoptosis in an animal model of pneumonia due to *Pneumocystis murina*, an infection localized to the lungs.

**Methods:**

Control mice and mice depleted of CD4+ lymphocytes were inoculated with *Pneumocystis*. Apoptosis of lung and spleen lymphocytes was assayed by flow cytometry and PCR assay of apoptotic proteins.

**Results:**

In control mice, apoptosis of lung lymphocytes was maximal just after the infection was cleared from lung tissue and then declined. However, in CD4-depleted mice, apoptosis was also upregulated in recruited lymphocytes in spite of progressive infection. In splenic lymphocytes, apoptosis was observed early at 1 week after inoculation and then declined. Apoptosis of lung lymphocytes in control mice was associated with a decrease in mRNA for Bcl-2 and an increase in mRNA for Bim. In CD4-depleted mice, lavaged CD8+ cells did change intracellular Bcl-2 but showed increased mRNA for Bim.

**Conclusion:**

Apoptosis of both pulmonary and extrapulmonary lymphocytes is part of the normal host response to *Pneumocystis *but is also triggered in CD4-deficient animals with progressive infection. In normal mice apoptosis of pulmonary lymphocytes may serve to terminate the immune response in lung tissue. Apoptosis of lung lymphocytes takes place via both the intrinsic and extrinsic apoptotic pathways and is associated with changes in both pro- and anti-apoptotic proteins.

## Background

Immune responses to an infectious pathogen must be tightly controlled to avoid excess inflammation and potential tissue injury, while still providing host defense and clearance of infection. This balance between beneficial and harmful inflammation is particularly critical in lung tissue, where the delicate alveolar capillary membrane responsible for gas exchange is easily disrupted by edema fluid and cellular injury. To protect lung tissue, the lungs are equipped with a variety of defense mechanisms which generally function to downregulate or suppress immune responses. For example, resident macrophages within the alveolar space function poorly as antigen-presenting cells, largely due to a lack of co-stimulatory molecules [1,2]. The fluid lining the alveolar space also functions to suppress lymphocyte proliferation and activation directly [3]. These factors and others make the alveolar space a difficult place in which to initiate an immune response to inhaled pathogens [4].

Nevertheless, when an infectious pathogen eludes phagocytosis by alveolar macrophages or mucociliary clearance, additional immune inflammatory cells must be recruited into lung tissue to prevent the infection from spreading. For many bacteria, these recruited cells are neutrophils as part of the innate immune response [5]. For other pathogens, effector lymphocytes are recruited into lung tissue as part of the adaptive immune response [6,7]. We and others have shown that lymphocytes are recruited into lung tissue via elaboration of specific chemokines [8,9]. However, mechanisms to turn off or limit lymphocyte recruitment, once the infection has been cleared, are poorly understood. Recruitment may be dampened through a loss of chemokine signals as the pathogen stimulus is lost. Alternatively, lymphocyte activation and proliferation may be suppressed directly though regulatory T-cells [10,11] or the elaboration of immunosuppressive eicosanoids [12,13]. An additional pathway to limit pulmonary immune responses is to shorten lymphocyte lifespan through apoptosis.

Apoptosis or programmed cell death is an important feature of embryogenesis, organ homeostasis, and hematopoiesis. Perturbations in apoptosis may lead to autoimmune disease or cancer. There are 2 main pathways for apoptosis in mammalian cells [14]: the Bcl-2 family regulated pathway (also known as the intrinsic mitochondrial pathway), mediated through cytokine receptors, glucose, and other stimuli and the TNF receptor (FAS) regulated pathway (also known as the extrinsic death receptor pathway). Both pathways involve activation of caspase enzymes-caspase 9 for the intrinsic pathway and caspase 8 for the extrinsic pathway with caspase 3 the final common death signal for both pathways. Apoptosis of lymphocytes has been proposed as a normal mechanism to turn off an immune response to antigen. After a lymphocyte response to antigen, most activated B and T cells die by apoptosis; the remaining cells form the basis of immunological memory [15]. Apoptotic cells display phosphatidyl serine on their surface which is the basis for the annexin assay, and this is recognized by macrophages for phagocytosis and removal of the apoptotic cells. In lung tissue, the percentage of apoptotic lung lymphocytes responding to intratracheal antigen (sheep red blood cell) increases after antigen exposure and then wanes, suggesting that apoptosis is part of the shut-off mechanism for an immune response [16]. In additional experiments using repeated intratracheal antigen challenge, lymphocyte apoptosis was upregulated, suggesting that apoptosis is a defense mechanism of the respiratory tract against serial antigenic challenges [17].

Lymphocyte apoptosis is also part of the systemic host response to infection, where it has generally been shown to be detrimental to the host by causing an immunosuppressive state. In mice transgenic for the anti-apoptotic protein Bcl-2, there is decreased lymphocyte apoptosis during sepsis and improved survival [18]. In murine models of sepsis, treatment with caspase inhibitors also increased survival [19]. Mice that are transgenic for the apoptosis inhibitory protein Akt are resistant to death from sepsis (cecal ligation/puncture) and there is less lymphocyte apoptosis [20]. Enhanced lymphocyte apoptosis has also been demonstrated in CD8+ T-cells from animals chronically infected with lymphocytic choriomeningtitis virus [21] and in mice infected with herpes virus [22]. In fact, some pathogens, such as *Listeria monocytogenes*, may stimulate lymphocyte apoptosis directly as a mechanism to evade host defenses [23].

Most of what is known about lymphocyte apoptosis in response to infection comes from models of systemic infection with viral or bacterial pathogens. Less is known about lymphocyte apoptosis during infection localized to a specific tissue or organ. The purpose of the present study was to characterize lung lymphocyte apoptosis during pulmonary infection with the fungal pathogen *Pneumocystis murina *(hereafter designated *Pneumocystis*). Pneumonia caused by *Pneumocystis *is restricted to the alveolar space (in murine models), and normal host defense requires recruitment of T-lymphocytes into lung tissue [7].

## Methods

### Animals

Specific pathogen-free BALB/c mice were purchased at 8 weeks of age from NCI/Charles River Breeding Labs (Wilmington, MA). Animals were housed in filter-topped cages and fed autoclaved chow and water ad libitum. Animals were injected intratracheally with *Pneumocystis *at a dose of 2 × 10^5 ^cysts per mouse.

Animals were sacrificed at 1, 2, 3 and 4 weeks after the inoculation. All caging procedures and surgical manipulations were done under a laminar flow hood. These experimental protocols were approved by the Institutional Animal Care and Use Committee at the Louisiana State University Health Sciences Center.

### *Pneumocystis *inoculation

*Pneumocystis *for inoculation was prepared as described earlier using lung homogenates from chronically infected *Scid *mice [24]. In brief, *Scid *mice chronically infected with *Pneumocystis *were injected with a lethal dose of pentobarbital. The animals were then exsanguinated by abdominal aortic transaction. The lungs were removed aseptically, placed in 1 ml of sterile PBS and then frozen at -70°C. Frozen lungs were homogenized mechanically in 10 ml of PBS by forcing tissue through a sterile 100 μm nylon strainer (BD Biosciences, Bedford, MA) and centrifuged at 1000 g for 10 min at 4°C. The pellet was resuspended in PBS. Dilutions (1:5 and 1:10) of this suspension were stained with Giemsa stain (Diff-Quick, Dade Behring, Newark, DE). The number of cysts was quantified microscopically and the concentration of inoculum was adjusted with PBS to 2 × 10^6 ^cysts/ml. Freshly prepared inoculum was always used for intratracheal inoculation to ensure the viability of organisms. Recipient mice were anesthetized with intraperitoneal injection (IP) of ketamine/xylazine (200 mg/kg and 10 mg/kg respectively). The trachea was surgically exposed. An 18-gauge blunt-ended needle was introduced into the trachea through the mouth under direct vision. *Pneumocystis *inoculum (2 × 10^5^*Pneumocystis *cysts in 0.1 ml) was injected through a 22-gauge inner needle into the lungs that was followed by an injection of 0.3 ml of air to ensure adequate dispersion of the inoculum and clearance of the central airways. The neck incision was sutured, and the mice were placed prone for recovery.

#### RNA isolation and real-time RT-PCR for PC rRNA

At animal sacrifice, total RNA was isolated from the right lung using TRIzol reagent (Invitrogen, Carlsbad, CA). cDNAs were synthesized from total lung RNA. As a standard for the assay, a portion of PC muris rRNA (GenBank Accession # AF257179) was cloned into PCR 2.1 Vector (Invitrogen, Carlsbad, CA) and PC rRNA was produced by *in vitro *transcription using T7 TNA polymerase (Promega, Madison, WI). TaqMan PCR primers for mouse PC rRNA were 5'-ATG AGG TGA AAA GTC GAAAGG G-3' and 5'-TGA TTG TCT CAG ATG AAA AAC CTC TT-3'. The probe was labeled with a reporter fluorescent dye, 6-carboxyfluorescein (FAM), and the sequence was 5'-6FAMAACAGCCCAGAATAATGAATAAAGTTCCTCAATTGTTACTAMRA-3' [25]. Real-time RT-PCR was done using a two step method. Reverse transcription reactions were done in a volume of 10 μl containing 200 ng RNA sample, 1 × TaqMan RT buffer, 5.5 mM magnesium chloride, 500 μM of each dNTP, 2.5 μM random hexamer, 0.4 U/μl Rnase inhibitor, 1.25 U/μl MultiScribe reverse transcriptase, (Applied Biosystems N 808-0234, Branchbug, New Jersey). Samples were incubated at 25°C for 10 min, reverse transcribed at 48°C for 30 min, reverse transcriptase inactivated at 95°C for 5 min. PCR reactions were done in a volume of 50 μl containing 5 μl (100 ng) cDNA, 1 × TaqMan universal PCR master mix (Applied Biosystems 4304437, Branchburg, New Jersey), primers and probe. An initial 2 min incubation was done at 50°C for UNG activity to prevent carryover reaction. The reaction was terminated by heating at 95°C for 5 min. The PCR amplification was performed for 40 cycles with each cycle at 94°C for 20 s and 60°C for 1 min. Data were converted to rRNA copy number using a standard curve of known copy PC rRNA and expressed as copy number per lung.

#### Depletion of CD4+ T-lymphocytes

In some experiments, mice were depleted of CD4+ T-lymphocytes by intraperitoneal injection of 0.3 mg of anti-CD4+ monoclonal antibody (hybridoma GK 1.5, ATCC) in 0.1 mi PBS each week. This treatment produces a sustained and profound depletion (always greater than 90%) of CD4+ lymphocytes from the blood and spleen allowing progressive *Pneumocystis *pneumonia [24]. We cannot rule out parallel depletion of CD4+ NK T-cells [26]. Depleted mice received a dose of anti-CD4+ antibody 3 days prior to *Pneumocystis *challenge and were then treated with antibody every 7 days.

#### Bronchoalveolar lavage (BAL)

Animals were sacrificed as described above. The trachea was exposed by a midline incision and cannulated with a polyethylene catheter. The lungs were lavaged with 10 ml of sterile Ca^2+ ^and Mg^2+^-free PBS in 1 ml steps. The first milliliter of BAL fluid was collected for cytokine assay. Cells were collected from the entire recovered BAL fluid by centrifugation at 300 g for 10 min at 4°C. Cell pellets were resuspended in PBS for counting in a hemacytometer and for flow cytometry analysis.

#### Collection of blood and spleen cell samples

A heparinized blood sample was obtained by cardiac puncture. Spleen was collected and teased apart in RPM-1640 (ATCC) medium. After centrifugation of the blood at 500 g for 10 min at room temperature, the plasma was collected and stored at -70°C for cytokine determination. Lymphocytes from the blood and spleen were enriched using Lympholyte-Mammal and Lympholyte-M (CEDARLANE. Burlington, NC) medium and procedures provided by the manufacturer. The contaminated red cells in the enriched lymphocyte fraction were lysed using RBC Lysis Solution (Gentra systems Minneapolis, MN). Blood leukocytes and BAL cells were counted using a light microscope with a hemacytometer. Differential counts were performed based on the morphological features of white blood cells stained with Giemsa stain (Diff-Quick, Dada Behring, Newark, DE). BAL cells and spleen cells (50,000 in number) were centrifuged (500 rpm for 5 min) onto glass slides, and stained with Giemsa stain for differential cell counting.

#### Flow cytometric analysis of lymphocyte apoptosis

Flow cytometric analysis was conducted on a FACSAria flow cytometer (BD Biosciences). Staining of cells for annexin-V, caspase 3 activity, caspase 8 activity, and caspase 9 activity were performed using a Vybrant Apoptosis Assay Kit #2 (Invitrogen), FAM-DEVD-FMK Caspase 3 detection kit, FAM-LETD-FMK Caspase 8 detection kit and FAM-LEHD-FMK Caspase 9 detection kit, respectively (Cell Technology, Mountain View, CA). The caspase assays label active caspases in living cells undergoing apoptosis [27]. Caspase detection using this methodology correlates well with other apoptosis assays and has been used by multiple investigators [28-30]. All cells were stained with optimal concentrations of fluorochrome-conjugated Abs specific for murine CD3 (BD), CD4 (Invitrogen) and CD8 (eBioscience), respectively. Isotype control antibody staining was used to assist in gating. It is difficult to conclusively label a cell as apoptotic, particularly in cells recovered from inflammatory sites. The best approach is to use more than one assay of apoptosis, as we have attempted to do here.

#### Isolation of CD4+ and CD8+ lymphocytes

Single-cell suspensions of BAL cells and spleen cells were incubated at 4°C for 15 min with optimal concentration of FcR Blocking Reagent as suggested by the manufacturer (Miltenyi Biotec). CD4 (L3T4) MicroBeads (Milteny Biotec, Auburn, CA) were then added to the cell suspension. After incubation at 4°C for 15 min, the cell suspension was run into a magnetic (MACS) column positioned against a permanent magnetic stand. The unlabeled cells which passed through the column were collected for the subsequent isolation of CD8+ lymphocytes. After washing the column three times with cold PBS, an appropriate amount of cold PBS was loaded onto the column. After separating the column from the magnetic stand, labeled CD4 enriched cells were flushed out from the column immediately. The procedure for isolation of CD8 cells from effluent cells from the CD4 column using CD8a (Ly-2) MicroBeads (Catalog No. 120-000-298) was the same as described for isolation of CD4 lymphocytes. Typical purity of isolated CD4+ and CD8+ lymphocytes was 85–90 percent.

#### Preparation of standard RNA for real-time RT-PCR of apoptosis protein mRNA expression

We chose to analyze mRNA for Bcl-2, Bim, and survivin because these gene have been implicated in lymphocyte apoptosis [31,32], but we acknowledge that apoptosis is complex and other genes may be equally important. Total RNA was isolated from lung tissue of an unmanipulated BALB/c mouse using Trizol reagent (Invitrogen). cDNAs for mBim (GeneBank accession no. AF032459) and mSurvivin (GeneBank accession no. NM 009689) were prepared by RT-PCR using the following primer pairs: mBim sense 5'-ATGGCCAAGCAACCTTCTG-3', mBim antisense 5'-TCAATGCCTTCTCCATACC-3'; mSurvivin sense 5,-ATGGGAGCTCCGGCGCTG-3', mSurvivin antisense 5'-CGATGTGGCATGTCACTCAG-3'CDNA for mBcl-2 was prepared from the pORF5-mBcl-2a general product (Invivogen). The cDNAs were cloned into PCR 2.1 Vector (Invitrogen) and the DNA sequencing was performed by Genomics Core Facility in LSU Health Sciences Center. RNA of mBcl-2, mBim and mSurvivin was produced by in vitro transcription using T7 RNA polymerase (Cat.# P 1300; Promega).

#### Real-time RT-PCR for apoptosis protein mRNA expression

Total RNA from CD4+ and CD8+ T-lymphocytes was isolated using the Versagene RNA purification kit (Fisher Scientific). Ten nanograms of total RNA was subjected to one-step RT-PCR using TaqMan RT-PCR reagents (Stratagene, La Jolla, CA) for murine Bcl-2 gene and two step RT-PCR using iQ Supermix kit (Bio-RAD) for murine Bim and Survivin genes. The real-time RT-PCR was determined on an iCycler thermocycler (Bio-Rad). Gene-specific primers and dual-labeled probe sequences for murine Bcl-2, Bim and Survivin mRNA and 18s ribosomal RNA (rRNA) were designed using Beacon Sesigner 2.12 (Premier Biosoft International) as follows (forward primer, reverse primer, and prober): mBcl-2, 5'-TGGGATGCCTTTGTGGAACTAT-3'; 5'-AGAGACAGCCAGGAGAAATCAAAC-3', 5'-TGGCCCCAGCATGCGACCTC-3'; mBim, 5'-AAACTTACACAAGGAGGGTGTTTG-3', 5'-AATGCCTTCTCCATACCAGACG-3', 5'-TTACCGCGAGGCTGAAGACCACCC-3'; mSurvivin, 5'-ATCGCCACCTTCAAGAACTGG-3', 5'-TCAGGCTCGTTCTCGGTAGG-3', 5'-ATGAAGCCAGCCTCCGCCATTCGC-3'; 18s rRNA, 5'-ATTCGAACGTCTGCCCTATCA-3', 5'-GTCACCCGTGGTCACCATG-3', 5'-TCGATGGTAGTCGCCGTGCCTACC-3'. All samples were normalized to 18s rRNA content. Data are expressed as transcript copy numbers per nanogram of 18s rRNA.

#### Immunohistochemistry

At 1, 2, 3, and 4 weeks after *Pneumocystis *infection, mice were sacrificed. Lungs were removed from each animal and inflated with 1 ml aqueous buffered zinc formalin (Z-Fix) (Cat# 175, Battle Creek, MI). The inflated lungs were then fixed in 20 ml of Z-Fix buffer. Fixed lungs were embedded in paraffin. Tissue blocks were sectioned at 4 μm thicknesses and slides were baked at 60°C for 45 minutes. Slides were deparaffinzed in Varistain 24-4 (Thermo Shandon, Ramsey, Minnesota) and target retrieval solution using a microwave pressure cooker. Slides were soaked in TBST buffer (Tris base 20 mM; Sodium Chloride 137 mM; and 0.1% Tween-20) for 1 minute and then in image iTFX Signal Enhancer (Cat#I36933, Carlsbad, CA) for 30 minutes to improve the fluorescence signal-to-noise ratio. Rabbit anti-Caspase 3 antibody (Cat# CP229 ABC, Biocare Medical, Concord, CA) and Rat anti-CD3 antibody were applied on to the slides and the slides were incubated at 4°C overnight. After washing 3 times with TBST, AF568-conjugated goat anti rabbit IgG antibody (Cat# A11036, Invitrogen; to conjugate rabbit anti-Caspase 3) and Alexa fluor 488 conjugated goat anti-rat IgG (Cat# A11006, Invitrogen; to conjugate rat anti-CD3) were added. The slides were incubated at room temperature for 5 min. After washing 3 times with TBST, DAPI, a nuclear stain emitting blue fluorescence upon binding to AT regions of DNA, was added on the slides. The slides were incubated at room temperature for 5 min. After washing with H_2_O, the slides were treated with Prolong Gold anti-fade reagent and then covered with coverslips in readiness for visualization under a LEICA DMRXA Deconvolution Microscope.

#### Statistics

Data are presented as mean + SEM. Sample size is indicated in each figure and table. Groups of 5–6 animals were studied at each time point, and all experiments were repeated at least twice. Data were compared by two-way analysis of variance followed by the Student-Newman Keuls test. Differences were considered statistically significant at *P *< .05.

## Results

### Pathogen burden following inoculation of *Pneumocystis*

**Figure 1:**
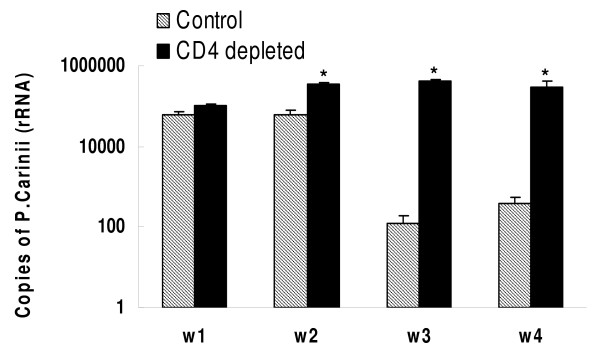
**Copies of *Pneumocystis *rRNA in the right lung of control and CD4 depleted mice infected with *Pneumocystis *for 1, 2, 3, and 4 weeks**. Note that the Y axis is a log scale. n = 5–6 for each time interval and for each condition (control, CD4 depleted). *: *P *< 0.05 vs control at same time point.

Normal mice housed in filter top cages have no detectable *Pneumocystis *rRNA in lung tissue. Control and CD4-depleted mice were inoculated with *Pneumocystis *and sacrificed at serial intervals after inoculation. Infection burden was assayed as copies of *Pneumocystis *rRNA in the resected right lung. (Previous experiments from our laboratory have shown no difference in infection burden in the right compared to the left lung within the same animal.) As shown in Figure 1, control mice inoculated with *Pneumocystis *showed an initial infection burden at 1 and 2 weeks after infection and then cleared the pathogen in that rRNA was no longer detectable at weeks 3 and 4. In contrast, mice with continuous depletion of CD4+ lymphocytes showed an increasing burden of infection that remained elevated at 4 weeks after inoculation. (Previous studies from our laboratory have shown that CD4-depleted mice will ultimately die of *Pneumocystis *pneumonia if depletion of CD4+ lymphocytes is maintained for greater than 8 weeks; they will clear the infection when treatment with anti-CD4 is stopped [24].)

### Recruitment of lymphocytes into lung tissue following inoculation of *Pneumocystis*

**Figure 2:**
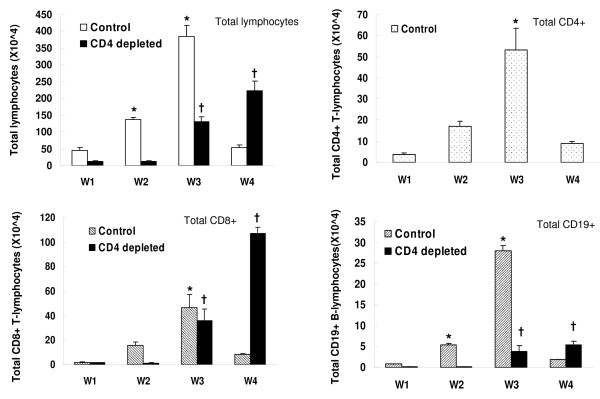
**Numbers of total lymphocytes, CD4+, CD8+ and CD19+ lymphocytes recovered in BALF of mice infected with *Pneumocystis *for 1–4 weeks**. n = 5–6 for each time interval and for each condition (control, CD4 depleted). *: *P *< 0.05 vs. W1 control; †: *P *< 0.05 vs. W1 CD4-depleted.

Lavaged lymphocytes were recovered at serial intervals from control and CD4-depleted mice after inoculation with *Pneumocystis *and analyzed for numbers of total lymphocytes, CD4+ lymphocytes, CD8+ lymphocytes, and CD19+ lymphocytes (Figure 2). Control mice began to recruit lymphocytes into lavage fluid by week 2 with a peak on week 3 and then a decline as the infection cleared. In contrast CD4-depleted mice showed a delayed recruitment of lavage lymphocytes to week 3 with continued increased lymphocytes at week 4. With regard to lymphocyte phenotypes, control mice recruited CD4+, CD8+, and CD19+ lymphocytes in a similar pattern (Figure 2). (For comparison, control mice without infection have essentially no lymphocytes in lavage fluid. Data not shown.) As expected, mice depleted of CD4+ cells did not have detectable CD4+ lymphocytes in lavage fluid at any time post inoculation of *Pneumocystis *(Figure 2). These mice were, however, able to recruit CD8+ lymphocytes and CD19+ B-lymphocytes into lavage fluid, again with delayed kinetics compared to control mice.

### Changes in spleen and blood lymphocyte numbers following inoculation of *Pneumocystis*

**Figure 3:**
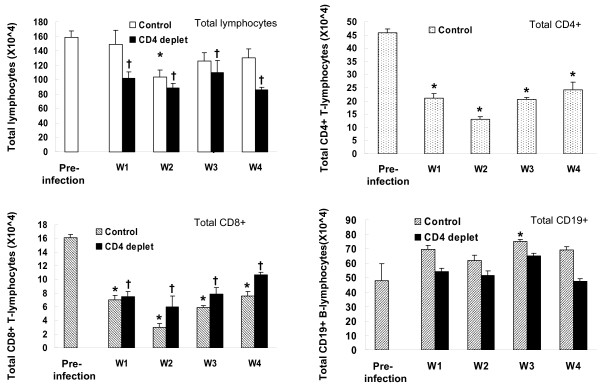
**Numbers of total lymphocytes, CD4+, CD8+ and CD19+ lymphocytes recovered from spleens of mice infected with *Pneumocystis *for 1–4 weeks**. n = 5–6 for each time interval. *: *P *< 0.05 vs. pre-infection; †: *P *< 0.05 vs. pre-infection.

Splenic lymphocytes were recovered at serial intervals from control and CD4-depleted mice after inoculation with *Pneumocystis *and analyzed for numbers of total lymphocytes, CD4+ lymphocytes, CD8+ lymphocytes, and CD19+ lymphocytes (Figure 3). Total lymphocyte numbers declined from normal on week 2 following *Pneumocystis *in control mice and then increased towards normal levels. In CD4-depleted mice, total lymphocyte numbers were decreased at week 1 and stayed decreased out to week 4. In control mice, CD4+ lymphocyte and CD8+ lymphocyte numbers were decreased at all times assayed post *Pneumocystis*, while CD19+ B lymphocytes showed a progressive increase in number following *Pneumocystis*. In CD4-depleted mice, CD8+ lymphocyte numbers were also decreased at all time points, while there was no significant change in the numbers of CD19+ lymphocytes. Note that CD4-depletion alone does not alter numbers of splenic CD8+ or CD19+ lymphocytes in mice [24].

In blood, there were similar changes observed after infection. There was an initial drop in total lymphocytes, CD4+ lymphocytes (except in the CD4-depleted group), CD8+ lymphocytes, and CD19+ lymphocytes on week 1 with a return towards normal levels by week 4. (Data not shown)

**Figure 4:**
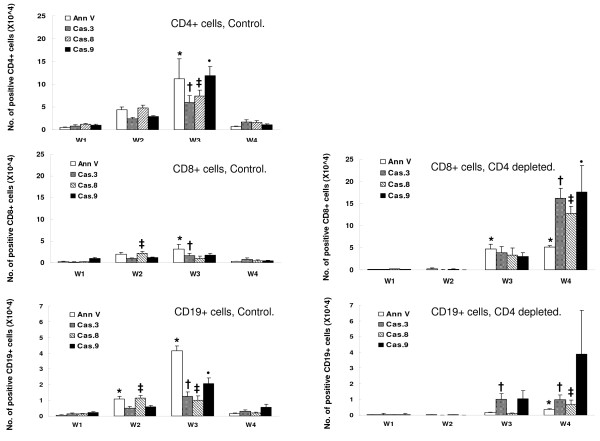
**Numbers of CD4+, CD8+ and CD19+ BAL lymphocytes with positive stain for annexin V, caspase 3, caspase 8 and caspase 9**. n = 5–6 for each time interval. *(annexin), †(caspase 3), ‡(caspase 8), and •(caspase 9): *P *< 0 .05 vs. week 1.

Apoptotic lung lymphocytes following inoculation of *Pneumocystis *Apoptosis of lavage lymphocytes was assayed by flow cytometry as surface staining of annexin V and intracellular activity of caspases 3, 8, and 9. In control mice, numbers of apoptotic cells began to increase in lavaged CD4+ lymphocytes by week 2, were significantly increased at week 3, and then declined to low levels (Figure 4). Note that maximal apoptosis of lung lymphocytes was at week 3 which was after the infection had been cleared from lung tissue (Figure 1). Changes in surface annexin staining and intracellular caspase activity did not always correlate, which we attribute to rapid clearance of annexin-positive cells by alveolar macrophages. Lymphocytes were often observed within alveolar macrophages in lung sections from infected animals.

In CD4-depleted mice, apoptosis was observed in recruited CD8+ and CD19+ lymphocytes beginning at week 3 and persisting to week 4 after *Pneumocystis *inoculation. The numbers of apoptotic CD8+ and CD19+ lymphocytes were greater than those observed in the control mice, and apoptosis ensued, even though the infection had not been cleared from lung tissue (Figure 1).

When lymphocyte apoptosis was expressed as a percentage of total cells rather than as total apoptotic cells, a similar pattern was observed. This data is presented in Table 1, Table 2, and Table 3.

**Table 1:** Percentage of apoptotic CD3+CD4+ cells

	Ann. V	Cas.3	Cas.8	Cas.9
Lavage	+CD4	+CD4	+CD4	+CD4

Week 1	8 ± 1	37 ± 5	50 ± 5	44 ± 5

Week 2	20 ± 3	12 ± 2†	28 ± 3‡	15 ± 1•

Week 3	21 ± 11	13 ± 4†	16 ± 5‡	24 ± 5•

Week 4	7 ± 1	18 ± 4†	36 ± 2‡	29 ± 4•

**Spleen**				

Pre-Infection	14 ± 3	8 ± 2	23 ± 2	21 ± 4

	13 ± 2	48 ± 3†	55 ± 2‡	48 ± 2•

	19 ± 5	6 ± 2	17 ± 4	3 ± 1•

	9 ± 1	5 ± 1	6 ± 1‡	13 ± 3

	4 ± 0	26 ± 4†	37 ± 8‡	29 ± 3

**Table 2:** Percentage of apoptotic CD3+CD8+ cells

	Ann. V		Cas.3		Cas.8		Cas.9	
Lavage	+CD4	-CD4	+CD4	-CD4	+CD4	-CD4	+CD4	-CD4

Week 1	5 ± 1	4 ± 0	25 ± 8	22 ± 8	34 ± 8	32 ± 12	31 ± 9	25 ± 12

Week 2	9 ± 2*	14 ± 3*	4 ± 1†	2 ± 1†	11 ± 2‡	6 ± 2	6 ± 2•	2 ± 0

Week 3	4 ± 1	8 ± 1	3 ± 1†	11 ± 2	2 ± 1‡	13 ± 5	4 ± 1•	12 ± 1

Week 4	3 ± 0	4 ± 0	6 ± 2†	8 ± 1	7 ± 3‡	16 ± 2	8 ± 3•	19 ± 4

**Spleen**								

Pre-infection	13 ± 4		7 ± 2		14 ± 2		11 ± 2	

Week 1	9 ± 2	10 ± 1	46 ± 4†	46 ± 3†	50 ± 2‡	57 ± 2‡	47 ± 2•	50 ± 2•

Week 2	15 ± 4	19 ± 1	4 ± 3	5 ± 3	6 ± 4	17 ± 7	2 ± 0•	1 ± 0

Week 3	7 ± 1	10 ± 2	4 ± 2	3 ± 0	2 ± 0	3 ± 1	3 ± 0•	5 ± 1

Week 4	4 ± 1	5 ± 0	24 ± 5†	11 ± 5	23 ± 9‡	16 ± 3	17 ± 3•	14 ± 4

**Table 3:** Percentage of apoptotic CD19+ cells

	Ann. V		Cas.3		Cas.8		Cas.9	
Lavage	+CD4	-CD4	+CD4	-CD4	+CD4	-CD4	+CD4	-CD4

Week 1	7 ± 1	8 ± 2	30 ± 8	27 ± 10	42 ± 9	38 ± 12	40 ± 12	36 ± 12

Week 2	18 ± 3*	32 ± 6*	10 ± 2	30 ± 12	20 ± 4	21 ± 6	12 ± 2	30 ± 9

Week 3	14 ± 1*	11 ± 2	7 ± 2	20 ± 4	7 ± 2‡	17 ± 6	11 ± 1	24 ± 5

Week 4	9 ± 1	7 ± 1	29 ± 13	14 ± 4	32 ± 13	26 ± 4	29 ± 11	38 ± 13

**Spleen**								

Pre-infection	33 ± 4		14 ± 3		21 ± 2		20 ± 4	

Week 1	26 ± 2	27 ± 4	53 ± 3†	50 ± 2†	59 ± 1‡	62 ± 3‡	55 ± 1•	57 ± 4•

Week 2	37 ± 9	37 ± 7	4 ± 2	4 ± 2	6 ± 2‡	14 ± 6	2 ± 1•	2 ± 1•

Week 3	19 ± 3	24 ± 3	4 ± 2	2 ± 0	4 ± 2‡	3 ± 2‡	9 ± 3•	5 ± 0•

Week 4	11 ± 2	16 ± 1	30 ± 5†	17 ± 6	29 ± 9	23 ± 3	26 ± 3	19 ± 4

**Figure 5:**
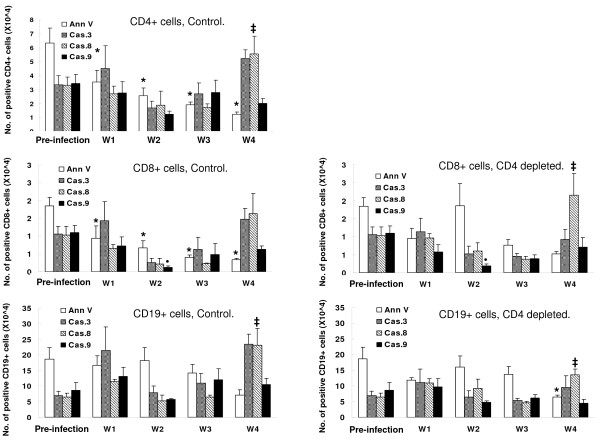
**Numbers of CD4+, CD8+ and CD19+ spleen lymphocytes with positive stain for annexin V, caspase 3, caspase 8 and caspase 9**. n = 5–6 for each time interval. *(annexin), †(caspase 3), ‡(caspase 8), and •(caspase 9): *P *< 0 .05 vs. pre-infection.

Apoptotic spleen lymphocytes following inoculation of *Pneumocystis*Splenic lymphocytes were isolated from control and CD4-depleted mice at serial intervals after inoculation of *Pneumocystis *and assayed for apoptosis (Figure 5). There was no consistent pattern of change in numbers of apoptotic splenic lymphocytes, though numbers of CD4+ and CD8+ cells with activated caspsase 3 and caspase 8 tended to increase on week 4. As with lung lymphocytes, we found that surface annexin and intracellular caspase activity did not always correlate, possible due to rapid phagocytosis and clearance of the annexin+ cells.

### Apoptotic proteins in lung and spleen lymphocytes following inoculation of *Pneumocystis*

**Figure 6:**
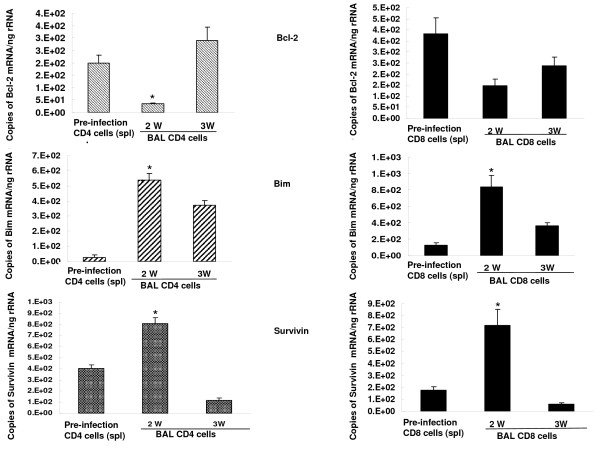
**Bcl-2, Bim and survivin mRNA expression in BAL CD4+ and CD8+ lymphocytes**. n = 5–6 for each time interval. *: *P *< 0.05 vs. pre-infection CD4 or CD8 cells (spleen).

Purified isolates of lung and spleen CD4+ and CD8+ lymphocytes were prepared at serial intervals after *Pneumocystis *inoculation of control mice and assayed for mRNA for the apoptotic proteins Bcl2, Bim, and survivin. Approximately 1 × 10^6 ^CD4+ lymphocytes and 0.5 × 10^6 ^CD8+ lymphocytes were recovered from total BAL cells (8–10 × 10^6 ^cells) of each mouse at 2 and 3 weeks following *Pneumocystis *inoculation. The results for BAL lymphocytes are shown in Figure 6. For BAL lymphocytes, comparison was made to mRNA for these proteins in CD4+ and CD8+ lymphocytes from control spleen, as control mice do not have sufficient BAL lymphocytes for analysis. At 2 weeks after *Pneumocystis*, lavaged CD4+ lymphocytes showed decreased mRNA for the anti-apoptotic protein Bcl2 and increased concentration of mRNA for the pro-apoptotic protein Bim (Figure 6, left hand column). Similar observations were made at 2 weeks in lavaged CD8+ lymphocytes with significant increased mRNA for Bim. Bcl2 mRNA was decreased at 2 weeks but did not reach statistical significance (Figure 6, right hand column). Survivin mRNA was increased in both BAL CD4+ and CD8+ lymphocytes at 2 weeks and then fell to control spleen levels.

**Figure 7:**
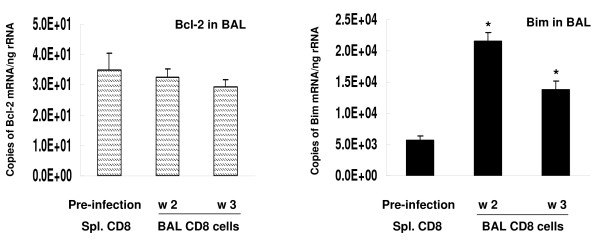
**Bcl-2 and Bim mRNA expression in BAL CD8+ lymphocytes from CD4-depleted mice**. n = 5–6 for each time interval. *:*P *< 0.05 vs. pre-infection CD8 cells (spleen).

In animals depleted of CD4+ cells, lavaged CD8+ lymphocytes showed no change from baseline in mRNA for Bcl-2 at 2 and 3 weeks after *Pneumocystis *(Figure 7). In contrast mRNA for Bim was increased in lavaged CD8+ cells at both time points.

**Figure 8:**
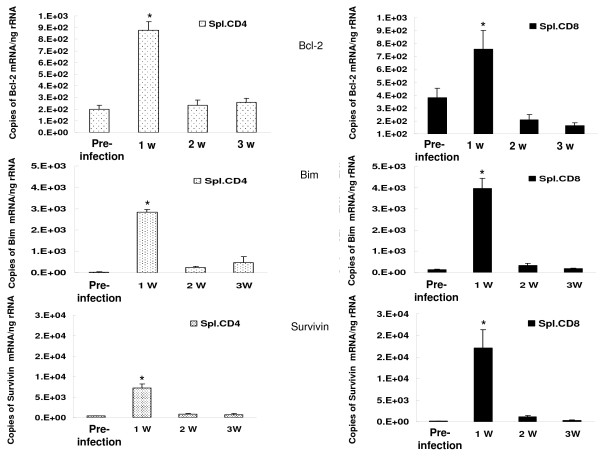
**Bcl-2, Bim and survivin mRNA expression in splenic CD4+ and CD8+ lymphocytes**. n = 5–6 for each time interval. *: *P *< 0.05 vs. pre-infection CD4 or CD8 cells.

We also assayed mRNA for apoptotic proteins in splenic CD4+ and CD8+ lymphocytes from control mice inoculated with *Pneumocystis *(Figure 8. In both CD4+ and CD8+ lymphocytes Bcl-2, Bim and survivin mRNA were increased above normal levels at 1 week and then declined. We did not observe reciprocal changes between Bcl-2 and Bim, as seen in lung lymphocytes however.

**Figure 9:**
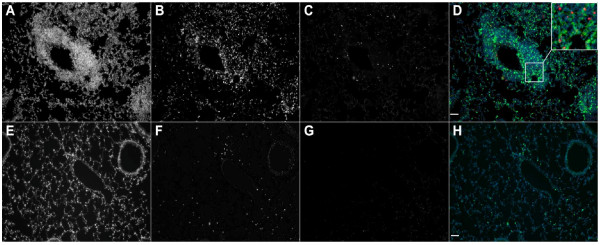
**Histological examination of lung tissue for CD3+ and caspase 3+ cells in mice 2 weeks post *Pneumocystis *infection (Panels A, B, C and D) and in control mice (Panels E, F, G and H)**. A/E: Caspase 3 and CD3 stains combined; B/F: CD3+ cells; C/G: caspase 3 + cells; D: Co localization of caspase 3 and CD3+ cells. Red color- caspase 3+ cells; Green color- CD3+ cells; Blue color- DAPI nuclear stain.

Tissue localization of apoptotic lymphocytes during infection with *Pneumocystis *Immunohistochemistry was employed to localize apoptotic lymphocytes in lung tissue of control mice 2 weeks after inoculation with *Pneumocystis*. We found that cells staining for CD3 (a T-lymphocyte marker) and for the apoptotic enzyme caspase 3 were rare in lung tissue from uninfected mice. However, in mice inoculated with *Pneumocystis*, there were significant accumulations of both CD3- and caspase 3-staining cells around pulmonary arterioles (Figure 9, Panels A and E). Combined staining showed that many but not all of the perivascular CD3+ lymphocytes were also caspase 3+ (Figure 9, Panels D and H). This perivascular location of recruited lymphocytes is consistent with previous results from our laboratory [33].

## Discussion

Apoptosis has been proposed as a mechanism to terminate a cellular immune response. According to this paradigm, once a pathogen has been eliminated, changes in the cellular milieu such as a fall in cytokine concentrations will initiate apoptosis of recruited lymphocytes and prevent tissue injury [34]. Although this is an appealing concept, almost all of the evidence in support of this idea comes from systemic infection with viruses [22] or systemic antigen challenge [35]. In experimental models of tuberculosis infection, lung lymphocyte apoptosis increases progressively, but the infection is never fully cleared [36]. In the current experiments we investigated apoptosis of lymphocytes recruited to lung tissue in response to localized infection with *Pneumocystis*, an infection that can be fully resolved in immunocompetent mice. Our results show that apoptosis of lymphocytes recruited in response to *Pneumocystis *infection begins in normal mice as soon as the lymphocytes enter lung tissue but is maximal at week 3 after the infection has been cleared and then declines. In CD4-depleted mice with progressive infection, on the other hand, apoptosis of CD8+ and CD19+ lymphocytes begins on week 3 when the infection has been established but then fails to decline. Our interpretation of these results is that apoptosis of recruited lymphocytes in control mice is triggered by clearance of the pathogen from lung tissue and is a probable host mechanism to terminate the cellular immune response and limit tissue inflammation. In CD4-depleted mice with persistent infection, the role of lymphocyte apoptosis is less obvious. Apoptosis of recruited lymphocytes increases progressively even though the infection is not cleared, and the numbers of apoptotic lymphocytes in lung tissue exceed those seen in control mice. In these immunodeficient animals, apoptosis may be controlled by activation programs of the individual cell (such as activation-induced cell death) rather than by extracellular factors that might influence the recruited lymphocyte population as a whole. The host defense role of pulmonary lymphocyte apoptosis in CD4-depleted mice infected with *Pneumocystis *is under investigation.

Apoptosis of cells can occur through two pathways, the intrinsic or mitochondrial pathways and the extrinsic death receptor pathway. Both pathways involve activation of caspase enzymes with effector capase-3 being the final common pathway [37]. In the intrinsic or mitochondrial pathway of cell death, apoptosis is triggered by damage to mitochondria resulting in an imbalance between anti-apoptotic Bcl-2 molecules and pro-apoptotic BH3-only molecules [14,32]. This imbalance results in activation of initiator capase-9 and cell death. The extrinsic apoptosis pathway is initiated by ligation of cell surface "death receptors" culminating in activation of initiator caspase-8 and cell death [38,39]. The results of the current experiments show that apoptosis of lymphocytes recruited to lung tissue in response to *Pneumocystis *involves activation of both caspase-8 and caspase-9. Thus, our results indicate that apoptosis of cells recruited to lung tissue involves both the intrinsic and the extrinsic pathways, at least for lymphocytes responding to *Pneumocystis*.

Apoptosis is regulated through an expanding number of intracellular proteins. Within the intrinsic apoptosis pathway, apoptosis is controlled through a balance or pro-apoptotic BH3-only proteins and anti-apoptotic Bcl-2 family proteins [14,32]. In addition, the protein survivin functions as an inhibitor of lymphocyte apoptosis [40,41]. In the present studies, we correlated lymphocyte apoptosis in lung tissue from control mice inoculated with *Pneumocystis *with messenger RNA for the BH3-only protein BIM, anti-apoptotic Bcl-2, and survivin. Our results show that apoptosis of both CD4+ and CD8+ pulmonary lymphocytes correlated with a drop in BCL-2 and a rise in BIM mRNA. These data are consistent with lymphocyte apoptosis being regulated through reciprocal interaction between these protein families. In contrast, we could not correlate pulmonary lymphocyte apoptosis with changes in survivin mRNA. Supporting data in survivin knock-out mice have shown that loss of survivin does not lead to lymphocyte apoptosis in vivo but is crucial to lymphocyte homeostasis and survival [42]. In splenic lymphocytes, all three apoptosis proteins were increased at 1 week, but we did not observe reciprocal changes in Bcl-2 and Bim for lymphocytes in this compartment. Investigation into how these proteins and other apoptotic proteins (PUMA, Bid, Bcl-xL) contribute to lymphocytes apoptosis in lung tissue is ongoing.

Apoptosis of lymphocytes in response to an infectious challenge could be both beneficial and harmful to the host. Benefits to the host could come from non-inflammatory removal of cellular debris, termination of the immune response (see above), and prevention of autoimmunity. Detriments to the host could come from abrogation of adaptive immunity and potential impairment of host defense against infection. With regard to this latter possibility, there is evidence in animal models that systemic infections causing the sepsis syndrome are associated with rapid onset of lymphocyte apoptosis in spleen and thymus [43,44]. Furthermore, inhibition of this apoptosis using caspase inhibitors [19] or transgenic animals lacking specific apoptosis-related molecules [20,31] improves survival in infected animals. Exactly how inhibition of lymphocyte apoptosis improves outcome in sepsis is not clear, but may involve preservation of lymphocyte-derived cytokines [19]. All of these observations have employed models of systemic infection. Even when lymphocyte apoptosis was studied in bacterial pneumonia [45,46], the models used were associated with bacteremia, indicating that the infection was not confined to the respiratory tract. In the current studies, we investigated whether *Pneumocystis *pneumonia, an infection confined to lung tissue, would also be associated with lymphocyte apoptosis in extrapulmonary organs. The results indicate that *Pneumocystis *infection, in a manner similar to systemic bacterial infections, also causes apoptosis of lymphocytes in the spleen. The mechanism(s) through which a pulmonary infection can trigger extrapulmonary lymphoid apoptosis are under investigation. It is not likely to be the result of endotoxin, as endotoxin assays of our *Pneumocystis *preps show very low levels (data not shown). It is possible that cytokine(s) release from pulmonary tissue triggers lymphocyte apoptosis in the spleen.

## Conclusion

We demonstrate in control mice that apoptosis of CD4+, CD8+ T-lymphocytes and B-lymphocytes in response to *Pneumocystis *infection begins with the onset of cellular recruitment into lung tissue but is maximal after the pathogen has been cleared, possibly serving as a mechanism to terminate the inflammatory response. In mice depleted of CD4+ lymphocytes, significant apoptosis of recruited CD8+ and B-lymphocytes is also observed even in the face of progressive infection and tissue inflammation. Apoptosis of lung lymphocytes in mice inoculated with *Pneumocystis *takes place via both the intrinsic and extrinsic apoptotic pathways and is associated with dysregulation of mRNA for pro- and anti-apoptotic proteins. Although the infection is localized to the lungs, there are associated changes in apoptotic proteins in splenic lymphocytes, suggesting that an apparently localized pulmonary infection can also stimulate lymphocyte apoptosis in extrapulmonary sites. These results indicate that apoptosis of pulmonary lymphocytes is part of the host response to infection with *Pneumocystis*.

## Competing interests

The authors declare that they have no competing interests.

## Authors' contributions

XS performed the animal studies and statistical analysis. NLC participated in the flow cytometry assays. XR assisted in the PCR assays. SR assisted with the apoptosis assays. JES conceived of the study and participated in its design and coordination. All authors read and approved the final manuscript.
